# Caregiving information needs of family caregivers of adolescent patients with suicide attempts: a qualitative study in China

**DOI:** 10.1186/s12912-024-02120-7

**Published:** 2024-06-28

**Authors:** Xiaoning Wang, Qunfang Miu, Jiannv Wang, Xiaoyu Huang, Wenru Xie

**Affiliations:** 1 School of Nursing, Hangzhou Normal University, Hangzhou City, Zhejiang Province China; 2 School of Clinical Medicine, Hangzhou Normal University, Hangzhou City, Zhejiang Province China; 3 Hangzhou Seventh People’s Hospital, Hangzhou City, Zhejiang Province China

**Keywords:** China, Attempted suicide, Adolescent, Information needs, Family caregiver, Stigma, Qualitative study

## Abstract

**Background:**

In the cultural milieu of China, family caregivers assume a pivotal role in the post-adolescent suicide attempt recovery journey. Nevertheless, they frequently encounter a dearth of requisite knowledge and information pertaining to the appropriate caregiving protocols for these adolescents. Notwithstanding, scholarly investigation into the informational requisites of this demographic concerning caregiving remains significantly constrained.

**Methods:**

Between September and December 2023, a phenomenological approach was applied in qualitative research. Semi-structured interviews were undertaken with 15 family caregivers of adolescents who had experienced suicide attempts. The amassed data underwent systematic organization and analysis through the utilization of the Colaizzi method.

**Results:**

Four primary themes were identified: (1) negative emotional encounters; (2) requirements for addressing dilemmas; (3) addressing the needs of the unknown; and (4) insufficient access to support.

**Conclusions:**

Family caregivers experience complex negative emotions upon learning about a teenager’s suicide attempt. Throughout the caregiving process, they face numerous challenges, with apparent lack of external support, leading to an increased urgent need for caregiving information. Healthcare professionals, especially nurses, should actively identify and respond to the informational needs of family caregivers when caring for adolescents who have attempted suicide. This includes providing education on various coping mechanisms and support strategies, as well as assisting them in better understanding how to effectively manage the stress and challenges of caregiving. By doing so, healthcare professionals can help alleviate the psychological and emotional burden on family caregivers, thereby enhancing their caregiving abilities and overall well-being.

**Supplementary Information:**

The online version contains supplementary material available at 10.1186/s12912-024-02120-7.

## Background

Attempted suicide refers to an individual’s intent to end their own life without resulting in death [1]. It is a pressing global public health issue and a leading cause of adolescent mortality. The United Nations Sustainable Development Goals (SDGs) and the World Health Organization’s Comprehensive Mental Health Action Plan for 2013–2030 have explicitly aimed to reduce global suicide mortality rates by one-third by 2030 [2]. Unfortunately, the incidence of attempted suicide among adolescents is currently increasing worldwide, with suicide ranking as the second leading cause of death among the 15–29 age group, only behind traffic accidents [3].

Across various countries, the stark reality of adolescent suicide attempts is evident. For example, a study in South Korea, which examined 468,482 adolescents, identified 15,012 cases (approximately 3.2%) confirmed as suicide attempts [4]. Similarly, in Poland, an investigation into 154 cases of adolescent suicide attempts showed that 64.3% were hospitalized after their initial suicide attempt, with males comprising 77.4% and females 61% [5]. In Chile, the prevalence of adolescent suicide attempts is 18.4% [6]. Meanwhile, in Colombia, a survey involving 32,076 adolescents aged 12 to 17 found that the prevalence of suicide attempts among females was 76.7%, significantly higher than that among males [7]. In China, a cross-sectional analysis of adolescent suicide attempts revealed an overall prevalence of approximately 2.94%, with rates of 2.5% for males and 3.17% for females [8]. These data underscore the severity of the issue of adolescent suicide attempts and indicate an increasing trend over time.

In China, suicide is often stigmatized due to societal and cultural norms, rendering it a sensitive topic that is rarely openly discussed [9]. This stance has resulted in widespread misunderstandings and silence regarding suicidal behavior and prevention methods, creating significant obstacles for caregivers in accessing relevant information and support systems. The stigma associated with suicide can lead caregivers to feel ashamed and hesitant when seeking assistance, amplifying their sense of isolation and stress in the caregiving process.

While the Chinese government and educational institutions have initiated efforts to provide mental health education and promote suicide prevention awareness, the dissemination and coverage of these initiatives remain limited [10, 11]. Further policy support and societal efforts are needed to ensure the accessibility of mental health resources and the effectiveness of suicide prevention measures. China’s family planning policy, also known as the one-child policy, has had profound effects on family structure and relationships among members. This policy has resulted in a reduction in family size, placing immense expectations and pressures on family members, especially the only child. Against the backdrop of intense educational competition, this pressure may escalate further, leading to increased mental health risks among adolescents and young adults. Furthermore, rapid social changes and economic transitions in China have significantly impacted individuals’ mental health. With increased social competition and a faster pace of life, individuals may experience heightened uncertainty and anxiety. Changes in family structure, such as rising divorce rates and the phenomenon of elderly people living alone, may also contribute to increased feelings of loneliness and psychological burden among family members.

During adolescence, teenagers undergo incomplete physical and psychological development, often lacking a precise understanding of suicidal behavior and possessing limited knowledge about suicide. Their access to accurate information on suicide is restricted, and they may struggle to recover effectively on their own, thus requiring external guidance. Adolescence is a pivotal stage in life, marked by various academic and interpersonal pressures.

In this demographic, there’s a higher prevalence of mental health issues like depression, anxiety disorders, and other psychological challenges, all of which are closely linked with suicidal behavior. According to statistics, 90% of suicide victims have been diagnosed with mental illness [12]. Various social factors, including conflicts within families, peer pressure, and bullying in school environments, can negatively impact adolescents’ mental well-being, further increasing the risk of suicidal tendencies. Additionally, cultural background, including societal attitudes towards suicide and the level of importance placed on mental health issues, also influence adolescents’ cognition and coping strategies regarding suicidal behavior. Economic status, such as financial difficulties within families or lower socioeconomic status in society, may exacerbate the stress experienced by adolescents, thereby increasing their risk of suicide. Health conditions, including chronic illnesses and disabilities, also affect adolescents’ sense of self-worth and life satisfaction, which may be related to the occurrence of suicidal behavior. Therefore, the interaction of multidimensional factors constitutes the complex background of adolescent suicidal behavior, necessitating comprehensive consideration of psychological, social, cultural, economic, and health-related factors to develop effective prevention and intervention measures.

The term ‘’family’’ encompasses diverse interpretations across cultures and societies but universally denotes an intimate social group comprising individuals with specific relationships, often rooted in blood ties, marriage, or legal adoption. Families play a pivotal role in the healthy development of adolescents, particularly concerning the prevention of suicidal behavior. The stability of the family environment, the quality of relationships among its members, and the level of communication significantly influence the psychological well-being and behavioral choices of adolescents. In this study, ‘’family caregivers’’ refers to those primarily responsible for tending to adolescents who have attempted suicide [13]. These caregivers may include parents, grandparents, siblings, or other legal guardians who offer emotional support and daily care to these adolescents. Family caregivers serve not only as sources of emotional support but also play a crucial role in preventing suicidal behavior and facilitating the recovery process of affected adolescents.

However, caring for individuals exhibiting suicidal tendencies presents an immensely daunting challenge. Present research predominantly centers on the needs of individuals with such tendencies, while the needs of family caregivers remain relatively underexplored. Family caregivers play a critical role in providing informational support, emotional comfort, and practical assistance, with their involvement being paramount in preventing suicidal behavior and promoting the psychological well-being of adolescents. Information needs encompass the sense of dissatisfaction and necessity for information experienced by individuals in real-life scenarios when grappling with various issues. The family constitutes the fundamental unit for the survival and development of adolescents, with family caregivers accompanying them through every stage of growth. These caregivers bear the responsibility of providing care, education, and medical rehabilitation pertinent to the development of adolescents with suicidal tendencies. They serve as a pivotal source of information support and wield a critical role in the recovery process of adolescents who have attempted suicide. As noted by Oexle [12], 90% of adolescents displaying suicidal behavior suffer from mental illness, often hesitant to communicate openly. This communication barrier creates an information gap between caregivers and the inner thoughts of the afflicted individuals, making family caregivers disadvantaged in accessing relevant information. Moreover, caregivers may experience additional stressors due to the ripple effects of adolescent suicide attempts, exacerbating their vulnerability. It is crucial for caregivers to obtain professional guidance to navigate this challenging phase, as neglecting to do so may compromise their well-being and impede the recovery of those affected. Currently, there is limited research in China on the information needs of caregivers of suicidal adolescents, highlighting the profound impact of stigma on this issue. Additionally, the effectiveness of various information acquisition methods directly influences both caregiver development and the recovery journey of the affected individuals.

In China, hospitals are the primary resource for suicide attempt survivors seeking help. Healthcare professionals act as intermediaries between patients and the social support system, offering prompt medical and psychological interventions to reduce the risk of further suicide attempts. Additionally, family caregivers involved in the process can gain valuable insights into caring for survivors. This study employs an interpretative phenomenological approach through in-depth interviews to fully grasp caregivers’ needs. This insight aids healthcare professionals in devising targeted information strategies, adopting a sensitive approach, and actively combating the stigma and misconceptions surrounding suicide, thereby providing more effective support to caregivers.

## Methods

### Study design and setting

The research has obtained approval from both the college and hospital ethics committees. This study employed an interpretive phenomenological approach within the qualitative research paradigm, to deeply capture and authentically reflect the unique psychological experiences and needs of caregivers when confronted with adolescent suicide attempts. Utilizing semi-structured interviews to explore the information needs of family caregivers of adolescents who have attempted suicide. This marks the first investigation of information needs among caregivers of suicide attempt survivors in China. The Consolidated Criteria for Reporting Qualitative Research (COREQ) checklist was employed to ensure methodological rigor in reporting qualitative findings [14].

### Sampling and sample size

Utilizing purposive sampling, family caregivers of adolescents who had experienced non-fatal suicidal behavior and were hospitalized in a psychiatric hospital in China between September and December 2023 were selectively identified. The chosen hospital is a large specialized psychiatric diagnostic and treatment institution in China, catering to patients with mental disorders from diverse regions across the nation.

The inclusion criteria were as follows: (1) Adolescents aged between 12 and 19 years old, meeting the diagnostic criteria for non-fatal suicidal behavior; (2) Caregivers identified as the primary family nurturers of adolescents, encompassing parents, grandparents, or other legal guardians; (3) No history of psychiatric disorders or significant cognitive impairments in both patients and caregivers, with unimpeded communication abilities. Exclusion criteria were delineated as follows: (1) Presence of severe physical illnesses and substance abuse; (2) Incapacity to collaborate in interviews or refusal to engage in the study due to various reasons. Every participant in this investigation willingly enrolled, and explicit informed consent forms were signed by both patients and their families. Data saturation was reached after conducting interviews with 15 family caregivers [15].

### Developing interview guidelines

After a comprehensive review of pertinent literature aligned with the research objectives, the research team, comprised of five members, meticulously crafted the interview guide. The team consists of an expert boasting over 30 years of clinical proficiency in psychiatric specialties, a director overseeing the psychiatric hospital nursing department with an extensive track record exceeding 20 years, a nurse manager contributing over a decade of experience in the child and adolescent ward, and two nursing master’s students possessing qualitative research expertise.

The initial interview guide underwent preliminary development, followed by pre-interviews with two family caregivers. Subsequently, the guide underwent meticulous refinement based on feedback, culminating in additional deliberations within the research group to craft the definitive interview guide. The inquiry encompasses the following aspects: (1) Kindly provide details regarding your child’s recent experience. (2) How did you emotionally respond upon discovering your child’s suicide attempt? (3) Throughout the caregiving process, what challenges and requirements have you encountered, and what are your primary concerns? (4) Along this journey, who has provided support, and in what specific ways has this assistance manifested? (5) In addition to the aforementioned questions regarding caregiving needs, are there any supplementary facets you wish to include or inquire about?

### Data collection

Detailed information regarding the research objectives, privacy protection, and other pertinent aspects was comprehensively communicated to all participants. Before their inclusion in the study, participants personally provided written informed consent. This investigation was undertaken during the patient’s inpatient care, subsequent to comprehensive communication with both the patient and their family beforehand. Employing a semi-structured, in-depth interview methodology, researchers meticulously scheduled interview sessions in advance with the participants.

Prior to the formal interviews, the research team delivered a comprehensive overview of the study’s objectives, expected interview duration, and privacy protection protocols. Informed consent was personally acquired, and participants formally endorsed the consent documentation during this direct, face-to-face interaction. The interviews transpired within a psychological counseling room meticulously selected for its serene ambiance, comfort, and heightened privacy. Researchers adeptly utilized a flexible interview framework, ensuring a seamless and organic conversational flow. Participants were prompted to spontaneously articulate their emotions, and caution was exercised to steer clear of leading or suggestive inquiries. With participants’ explicit consent, the interviews were recorded, accompanied by scrupulous note-taking to capture the nuances of the discussions. In instances where information appeared unclear, additional inquiries were made to ensure thorough clarification. The duration of patient interviews ranged from 30 to 40 min, and respondents were identified using the anonymous code ‘’N’’. After each interview concluded, researchers expeditiously transcribed the audio recordings into textual data. Subsequently, these transcriptions were intricately organized in collaboration with interview notes. The consolidated data were then shared with the interviewees for validation and feedback.

### Data analysis

Within 24 h of concluding the interviews, the interviewer diligently transcribed the recorded content into written documentation. Following this, two researchers embarked on a meticulous and iterative analysis of the identical data set. Their process involved a consistent and thorough comparison of their findings with the original material, closely examining the rationality and logic embedded in the data. The analysis strives to steer clear of integrating the researcher’s individual theories and experiences, aiming for a state of suspension [16]. Consistent reflection, immersion, and exploration of data themes are actively pursued to uphold quality control standards. Systematic classification and coding are facilitated through the utilization of Nvivo 1.2 software, while the application of the Colaizzi 7-step analysis method enhances the precision of data analysis [17].

The specific steps are as follows: (1) Thoroughly review all interview records; (2) Extract key statements; (3) Code recurring viewpoints; (4) Summarize and synthesize coded viewpoints into themes; (5) Provide a comprehensive description of the refined themes; (6) Identify similar viewpoints and refine thematic concepts; (7) Promptly provide feedback to participants for verification and validation. Upon theme confirmation, cross-check with participants. Any inconsistencies prompt theme re-extraction, resulting in final data. Throughout, anonymization ensures researcher impartiality.

Following comprehensive analysis, various themes were systematically categorized into relevant domains, encompassing aspects such as emotional care, challenging caregiving, care needs, caregiving anxiety, and inadequate support. Disputes were resolved through discussion and analysis with a third researcher until a consensus was reached. During this process, recognizing the similarities and overlap between challenging caregiving and care needs, the care needs were amalgamated into the category of challenging caregiving. The final thematic divisions comprised four principal sections.

## Results

### Demographics

Fifteen family caregivers were interviewed, coded as N1 to N15. Table 1 summarizes the basic characteristics of the participants and their adolescent patients.

### Emergent themes and sub-themes

The study identified four primary themes with eight corresponding subthemes. Notably, the fourth theme, ‘’Inadequate Social Support’’ does not incorporate additional subthemes due to insufficient information. The subsequent section presents a more in-depth analysis of each theme, including negative emotional encounters, requirements for addressing dilemmas, addressing the needs of the unknown and insufficient access to support. Please refer to Table 2 for a breakdown of the thematic analysis.

**Table 1:** General Information of Pediatric Patients and parents (*N* = 15)

Number	Parents of the Child				Child				
	Relationships	Age (years)	Educational Level	Occupation	Age (years)	Gender	Disease Diagnosis	Suicide Method	Number of Suicide Attempts
N1	Grandmother	62	junior high school	unemployed	15	female	depression	jumping off a building	1
N2	Father	46	Elementary School	Worker	16	female	depression	taking pills	1
N3	mother	48	Elementary School	Worker	19	male	depression	cliff jumping	1
N4	mother	36	High School	staff member	14	female	depression	wrist cutting	1
N5	Father	47	University	Lawyer	17	female	depression	taking pills	1
N6	Grandmother	65	Elementary School	Cleaner	17	male	obsessive-compulsive disorder	wrist cutting	1
N7	Sister	28	High School	Accountant	16	male	depression	taking pills	1
N8	mother	35	University	staff member	14	female	depression	taking pills	1
N9	Father	49	Elementary School	farmer	19	female	auditory hallucination	jumping off a building	1
N10	mother	39	High School	Worker	14	female	depression	taking pills	1
N11	mother	40	High School	logistics	14	female	depression	jumping off a building	1
N12	Father	40	junior high school	Worker	14	female	depression	taking pills	1
N13	mother	45	High School	Preschool Teacher	17	female	depression	taking pills	1
N14	mother	43	junior high school	Worker	15	female	depression	taking pills	1
N15	mother	50	High School	staff member	15	male	depression	jumping off a building	1

**Table 2:** Overview of the themes and associated sub-themes

Main themes	Sub-themes
Negative emotional encounters	Feelings of suspicion and distress
	Fear and helplessness
	Self-blame and regret
Requirements for addressing dilemmas	Abnormal behavior
	Repeated self-harm
	Communication difficulties
Addressing the needs of the unknown	Concerns about further suicide
	Concerns about future life
Insufficient access to support	

### Negative emotional encounters

#### Feelings of suspicion and distress

In the face of the tragedy of a child’s suicide, family caregivers commonly experience profound confusion and incredulous emotions. The suddenness and unpredictability of suicidal behavior make it difficult for family caregivers to accept this reality initially. The psychological state and responses of family caregivers are multifaceted, involving a reevaluation of the value of life, an understanding of mental health issues, and a reflection on family relationships and support systems.

Caregivers were extremely shocked upon hearing the news of their child’s suicide, finding it difficult to believe that the child would choose such an act.

*I wasn’t the first to know; when the child told me, I couldn’t believe it, feeling that she wouldn’t engage in such extreme behavior. (N1)*

*She told me she took pills, and I still couldn’t believe it. A teenager, what could be the matter? (N2)*

The profound shock and reluctance to acknowledge reality experienced by family caregivers in the face of sudden tragedy are evident. Moreover, when a child’s condition shows signs of improvement only to relapse into suicidal behavior, it presents an exceedingly challenging scenario for caregivers to grasp and accept.

*Called around 11 in the morning saying she took pills. I certainly didn’t want to believe it because it seemed like the child was getting better; it couldn’t suddenly be like this. (N10)*

This reflects the confusion and helplessness experienced by family caregivers when faced with the reality of a child’s suicide. Caregivers often avoid delving into the underlying reasons for the child’s suicide, possibly due to fear of confronting painful truths or a lack of understanding of mental health issues. For instance, a family caregiver initially attributed a child’s medication after staying up late to mere tiredness, without immediately realizing the severity of the problem.

*She swallowed the pills at eleven at night, couldn’t be awakened the next morning, still couldn’t be awakened by noon. I thought he was just too tired, stayed up too late. Later, seeing pills scattered around him, I dared not think in that direction, never thought it would be like this. (N7)*

The psychological process of family caregivers transitions from shock and denial to confusion. These narratives reveal the complex emotions and psychological states experienced by family caregivers when confronted with a child’s suicide. It is evident that family caregivers undergo inner turmoil and emotional conflict in dealing with this tragedy.

### Fear and helplessness

Family caregivers commonly experience intense panic and uncertainty when faced with an emergency situation involving their child’s suicide attempt. The emergence of these emotions is primarily due to fear of the potential ineffectiveness of intervention and concerns about unknown consequences. In such situations, caregivers must quickly recover from a state of shock and mobilize their rationality and composure to deal with the crisis. For example, a caregiver’s reaction upon learning of their child’s medication intake reveals this psychological state:

*Upon learning that the child had ingested pills, I was terrified, completely shattered. There was a fear that those pills might be something else—what if? I hesitated for a moment, but quickly jolted myself awake. (N8)*

In the process of taking action, despite being filled with fear, caregivers still need to make decisions swiftly. This decision-making process reflects the caregivers’ rational responses in emergency situations.

*I felt an intense fear in that moment, unsure of the next steps. I stood there in a daze for what felt like an eternity before regaining my composure and rushing to the hospital. (N12)*

Even after their children receive emergency treatment, caregivers’ sense of helplessness is not completely alleviated. They continue to face profound psychological challenges throughout their children’s treatment process.

*I didn’t see the child initially; they were already in the emergency room. Standing at the entrance, I felt a deep sense of helplessness and unease. What if we couldn’t bring them back? What purpose would my existence serve? (N15)*

Family caregivers, when faced with their child’s suicide attempt, undergo a continuum of psychological changes from initial panic and collapse to swift responses, and then to feelings of helplessness and reflection during the treatment process. This process not only reveals the caregivers’ emotional fluctuations but also underscores their psychological coping strategies in crisis. The initial panic demonstrates their immediate reaction to the event, while the rapid response during emergencies showcases their ability to mobilize rationality. The feelings of helplessness and unease during the treatment process further reveal their emotional adjustment amidst ongoing challenges.

### Self-blame and regret

When witnessing a child’s self-harming behavior, caregivers often experience intense feelings of self-blame. This self-blame stems from directly witnessing the child’s pain and empathizing with their expressed desire to end their life. For example, one caregiver expressed their feelings when facing their child’s distress:

*Her desire to die…crying… I haven’t adequately cared for my granddaughter. If, in the end, she succumbs to the fate of those cases we see online (suicidal), then what purpose does my existence serve? (N1)*

This sense of self-blame extends further to question the caregiver’s own role. Family caregivers may reflect on their absence during the child’s upbringing and the fact that, due to busy work schedules and other reasons, they couldn’t provide adequate attention and companionship to the child. They believe they failed to offer necessary support and guidance during crucial stages of the child’s personality development, resulting in an undeniable sense of responsibility. One caregiver articulated this feeling:

*During the critical phase of shaping the child’s personality, we were not present by her side. I find it challenging to accept the way the child has grown and the development of her personality. We’ve been engrossed in our work, offering scant attention to the child. In a year, we haven’t even dedicated a third of our time to her. The child has evolved in this manner, and the responsibility lies solely with me. (N5)*

As the primary caregivers for the child, they also experience profound feelings of powerlessness and confusion when confronted with the child’s behavioral changes. This emotional experience involves reflecting on past actions, feeling helpless in the current situation, and worrying about potential future outcomes.

*I’ve been the primary caregiver for this child since her early years. Now that she has undergone this transformation, there are aspects I find difficult to articulate. (N6)*

Furthermore, family caregivers may become aware of their shortcomings in cultural literacy, which further intensifies their self-blame and regret. They believe they haven’t done enough to provide appropriate education and guidance to the child, deepening their sense of responsibility.

*My cultural literacy is notably insufficient. I genuinely feel that I’ve fallen short in providing the child with a proper education. (N9)*

The psychological experiences of family caregivers following a child’s suicide attempt are complex and profound. Their feelings of self-blame and regret encompass not only reflections on past actions but also questioning their own roles and responsibilities, as well as self-assessment of cultural literacy and educational approaches.

### Requirements for addressing dilemmas

#### Abnormal behavior

When caregivers discover that their children are smoking, they initially experience a conflict between concern and responsibility. They realize that this behavior may have negative effects on their child’s health, while also sensing the psychological pressures behind the child’s attempt to conceal the behavior. As one caregiver mentioned:

*I uncovered a rather concerning habit of hers – smoking. Initially, she attempted to conceal it, but her cousin witnessed it and informed me, revealing the truth. Now, I’m hesitant to allow her emotions to sway freely. (N5)*

This discovery triggers caregivers to deeply contemplate how to balance acceptance with guidance. Faced with this challenge, caregivers seek a way to express both emotional understanding and respect for their children, while also conveying expectations for a healthy lifestyle. The inner conflict they experience is reflected in statements such as:

*I reassure her, saying, ‘There’s no need to be so apprehensive; it’s alright to smoke in my presence. ' However, I genuinely struggle to accept my daughter smoking. To be candid, I am internally conflicted, uncertain of how to provide her with guidance. (N5)*

Here, caregivers seeking a balance between acceptance and guidance reflect a complex psychological state that considers both emotional freedom and behavioral consequences. When it comes to their child’s sexual orientation, caregivers also face a conflict between emotional acceptance and societal expectations. The child’s honesty not only surprises caregivers but also compels them to reevaluate their understanding and level of acceptance regarding sexual orientation. A caregiver’s reaction to their child coming out as liking the same sex might be:

*During the high-speed train journey here, she confided in me, saying, ‘Mom, I like a girl. ' Initially, I considered it normal, but the depth of her affection caught me off guard. I remained silent at the time. I grapple with the dilemma of guiding her on the appropriateness of her feelings. I expressed concern that if it’s not what she genuinely desires, might she act impulsively again? (N14)*

This silence and perplexity reflect the caregiver’s endeavor to respect the child’s self-identity while exploring how to offer appropriate life guidance. The caregiver’s experience in this process transcends mere reactions to singular events; rather, it entails profound contemplation of the family’s values, parent-child relationships, and educational approaches. Caregivers must provide support and guidance based on an understanding of the child’s personality and needs, while simultaneously managing their own emotions and expectations. This experience demands caregivers to demonstrate a high degree of sensitivity, adaptability, and educational wisdom.

### Repeated self-harm

Repetitive self-harm behaviors in children present a serious and complex mental health issue, placing significant psychological burdens on family caregivers. They strive to find a balance between understanding the child’s internal needs and providing effective interventions. One caregiver described this dilemma as follows:

*Frequently, she expresses a desire to jump off a building and resorts to using a small knife to harm herself. Attempts to intervene prove futile, and I only become aware of her self-inflicted wounds upon noticing the scars on her arms. Despite my conversations with her, urging her to refrain from such actions, she agrees momentarily, only to resume cutting a few days later when experiencing mental unease. (N2)*

*They perceive it as a bold and trendy form of expression, and she mimics it. I’m at a loss on how to counsel the child. (N9)*

While caregivers seek to understand the psychological motivations behind a child’s self-harm behaviors, they also endeavor to find appropriate intervention strategies. They attempt to provide alternative coping mechanisms, such as encouraging the child to seek other outlets during emotional fluctuations. However, these efforts often struggle to sustain effectiveness.

*On one occasion, she cut too deeply, witnessed blood, felt frightened, promptly sanitized and bandaged herself. However, in subsequent emotional downturns, she resorts to cutting again. I advise her that it’s acceptable to vent on a pillow, encouraging her not to harm herself, but my efforts prove ineffective. (N12)*

When dealing with a child’s self-harm behaviors, caregivers also face challenges related to values and educational approaches. They need to find an appropriate balance between respecting the child’s autonomy and ensuring their safety. One caregiver expressed this dilemma:

*I implored her to cease cutting, to which she responded, ‘Seeing blood excites me, liberates me, makes me happy. I must cut. ' Despite removing all sharp objects from her room, the situation did not improve. (N13)*

Caregivers encounter obstacles when trying to understand the motivations behind a child’s self-harm behaviors. Their psychological experiences are multidimensional, requiring them to manage their own emotional responses and provide appropriate support and guidance to the child in the absence of mental health knowledge. This experience demands caregivers to demonstrate empathy, patience, and adaptability.

### Communication difficulties

In the family setting, communication serves as a crucial link for maintaining relationships among members and understanding individual needs. However, when children face mental health challenges, communication with them often becomes complex and difficult. Family caregivers frequently encounter formidable obstacles in their attempts to establish effective communication with the child. These obstacles arise not only from the child’s psychological state but also from the caregivers’ own communication skills and emotional resilience.

Family caregivers face challenges in communicating with children, primarily evident in the difficulty of understanding the child’s inner world. Children may struggle to open up to caregivers due to various reasons such as psychological disorders or emotional distress. This communication breakdown leaves caregivers feeling helpless and anxious as they desire to comprehend the child’s true feelings and provide appropriate assistance. For instance, a caregiver expressed such concerns:

*My primary concern now is that my child doesn’t open up to me; there’s a communication gap. I struggle to understand her, and I sincerely hope that, regardless of the issues she’s facing, she would engage in a meaningful dialogue with me so that I can find ways to offer assistance. (N9)*

Furthermore, even in cases where communication frequency has increased, the quality of communication may not necessarily improve. Children may still be unwilling to share their feelings and experiences, making it difficult for caregivers to find a point of entry to offer assistance. As described by another caregiver:

*Our communication with the child has increased since she fell ill, but even then, we can’t engage in a substantial conversation. She doesn’t share details about her recent situation with us, and we’re unsure about how to proceed without causing distress. (N13)*

Caregivers may also encounter direct resistance and rejection from the child when attempting to communicate. This reaction further exacerbates the difficulty of communication, leaving caregivers feeling confused and frustrated in their search for effective communication methods. As described by one caregiver:

*I find myself either going along with or comforting her because, at times, if I say one more thing, she’ll respond with, ‘Oh, stop it. I don’t want to hear your motivational talk. Just be quiet! ' That’s how straightforward she can be. (N15)*

This highlights the challenges that family caregivers face when communicating with children, as well as their relentless efforts to establish effective communication.

### Addressing the needs of the unknown

#### Concerns about further suicide

In the home environment, the recurrence of suicidal behavior in children is a central concern for caregivers. This concern not only involves a direct threat to the child’s life safety but also relates to the overall psychological stability and emotional well-being of the family. Faced with this issue, caregivers often find themselves in a state of uncertainty and helplessness, urgently seeking effective prevention measures and coping strategies.

Caregivers, when dealing with this issue, first and foremost experience a profound sense of uncertainty and deep concern regarding the future behavior of the child. They realize that despite possibly being physically close to the child, they cannot fully comprehend the child’s inner world and behavioral tendencies. This uncertainty renders caregivers feeling powerless in preventing suicidal behavior.

*The thought of my child attempting suicide again worries me deeply. How can I prevent it? Even if I’m home every day, I remain unaware of her activities in her room; I can’t gain access. Sigh… (N12)*.

Furthermore, caregivers may directly confront the child’s attitudes and feelings towards suicidal behavior during communication. This direct interaction often brings significant emotional impact and psychological burden to the caregivers.

*After some improvement during that time, I asked him, ‘Would you regret this?’ He replied, ‘Even if there’s a next time, I’d still do it… ‘ (choked up) I’m genuinely scared that, someday, he might… accidentally, you know… What should I do? (N15)*

Caregivers, when facing the risk of recurrence of suicidal behavior in children, not only need to manage their own emotional reactions but also to provide appropriate support and guidance to the child in the absence of sufficient mental health knowledge. This experience requires caregivers to demonstrate a high degree of empathy, patience, and adaptability.

### Concerns about future life

Family caregivers often face dual concerns regarding the mental health and life trajectory of their children when considering their future. They feel uneasy about the long-term effects of the child’s ongoing suicidal behavior, stemming from a profound understanding of the child’s future societal role and functioning.

Caregivers express profound concern about their child’s ability to live independently in the future. They question how the child will achieve independent living and navigate life’s path in the face of incurable underlying illnesses. This concern is reflected in the words of one caregiver:

*Is it possible to eliminate the underlying cause of his illness? If the root cause proves incurable, how should life be navigated in the future? Can he attain independence? (N3)*

Simultaneously, caregivers express clear concerns about their child’s employment prospects. They worry that the child’s suicidal behavior could become a barrier to future employment, affecting their ability to lead a life comparable to that of ordinary individuals. This concern is exemplified in the narrative of another caregiver:

*My primary concern is whether my child’s suicidal behavior will impact her future employment. Will she be able to lead a life comparable to that of an average individual? (N10)*

Furthermore, caregivers are aware that certain career paths, such as military service, may no longer be attainable for their children. This realization brings additional challenges:

*I acknowledge that, having undergone hospitalization here, pursuing a military career in the future is undoubtedly unattainable. (N13)*

Caregivers also express concerns about the possibility of their children inheriting or developing mental illnesses similar to those present in other family members. This further exacerbates their pessimism about the future of their children.

*I worry that he might face mental instability similar to that of his paternal uncle’s after going through all of this. (N15)*

This reveals the profound concerns of family caregivers regarding the future life of their children. These concerns not only involve caring for the child’s personal happiness but also encompass the impact on their social functioning and career development. The experiences of family caregivers in this process are complex and profound, requiring us to understand and support them in a more detailed and empathetic manner.

### Insufficient access to support

Family caregivers perceive seeking help at the hospital as their only feasible option to avoid potential negative judgments and adverse effects on their child’s development. For example, one caregiver expressed concerns about the consequences of disclosing information publicly:

*It would not be ideal if the neighbors became aware; it could present challenges for this child as she matures! Currently, she is not attending school, and her seclusion goes unnoticed by others. The hesitancy to go outside is influenced by the presence of numerous neighbors. If someone spots her and inquires, how do we navigate that conversation? (N1)*

This reflects their tendency to maintain the child’s withdrawal status and avoid external interference. In specific regions, family caregivers face significant socio-cultural pressures, fearing that having a child with mental health issues at home might alter people’s perceptions of them. Hence, they are more inclined to seek help at the hospital rather than openly discuss the child’s situation. One caregiver pointed out:

*There is limited assistance available in other aspects. Seeking help at the hospital remains the sole viable option! In our region (Xinjiang), having a child like this at home alters people’s perceptions. I would rather not expose her to such an environment.(N5)*.

This reveals the socio-cultural pressures that family caregivers encounter when seeking help. Family caregivers also face challenges when discussing the child’s situation, fearing that such discussions might burden the child further. For example, one caregiver mentioned: *We have a relative who took their own life on the 30th day of the lunar new year due to the same illness (depression). Despite it being several years, we only discovered the reason after their passing. We don’t wish to disclose too much about our child’s situation; our primary goal is to come to the hospital to assist her in emotional adjustment. (N7)*

This indicates that family caregivers need to weigh the pros and cons of open discussions when considering how to address their child’s mental health issues. Additionally, family caregivers protect their children from social stigma by concealing information. One caregiver shared a story of a relative who committed suicide due to depression and expressed: *Discussing the child’s situation with outsiders is challenging; it would burden her. If we mention her hospitalization at the Seventh People’s Hospital (a local psychiatric facility), how would people interpret it? Suicide? Mental health issues? The potential judgments are concerning. (N6)*

This reveals the strategies family caregivers employ in dealing with their child’s mental health issues, using secrecy to avoid potential negative societal judgments.

## Discussion

Adolescent suicide attempts are a pervasive global mental health concern, where family caregivers play a pivotal role in preventing further incidents. However, grappling with a teenager’s suicide attempt becomes a traumatic event for these caregivers, triggering intricate emotional responses. The study by Maple and colleagues suggests that family caregivers experiencing prolonged high levels of stress and emotional challenges may be at risk of severe psychological distress [18]. This finding aligns with the results of our study. Caregivers are not immediately aware of a child’s suicidal behavior. Upon notification, they initially respond with suspicion, followed by panic, and ultimately, self-blame. Emotional transitions range from struggling to accept the situation to being immersed in self-reproach. Caregivers managing adolescents who have attempted suicide endure significant emotional strain, to the extent that they may even contemplate thoughts of death themselves. In this study, it appears that caregivers were informed of the adolescent’s suicidal behavior by the adolescents themselves. This may be influenced by the following factors: (1) Communication barriers: In many Chinese families, particularly in more traditional ones, intergenerational communication barriers may exist. There could be a lack of open and honest emotional communication between elders and adolescents, leading adolescents to opt for coping with psychological distress alone rather than seeking help from family members [19]. (2) Neglect of mental health: Traditional Chinese culture may prioritize academic achievements and social status over mental well-being, resulting in caregivers potentially overlooking psychological issues in adolescents and lacking the knowledge to identify and address them [20]. (3) Stigma: Mental health issues in some cultural contexts are often associated with stigma, leading adolescents to fear being labeled or discriminated against. As a result, they may opt to conceal their suicidal thoughts and behaviors from caregivers to avoid disclosure [21]. (4) Lack of recognition of warning signs: Family caregivers may lack training in identifying the warning signs of suicidal behavior in adolescents, such as mood changes, unusual behaviors, or verbal cues. (5) Family structure and function: Differences in family structure and functionality can also affect interactions and communication among family members. In certain households, the absence of effective support systems and problem-solving mechanisms may leave adolescents without timely assistance when confronting stress and challenges. (6) Cultural values: Some aspects of traditional Chinese culture, such as emphasis on filial piety, harmony, and conflict avoidance, may deter adolescents from expressing negative emotions to family members to prevent family distress.

A qualitative study on the reconstruction of parental identity demonstrated a deficiency in caregivers’ understanding of the factors influencing adolescent suicidal behavior [22]. Compounded by being entwined in negative emotions, caregivers find themselves unable to offer beneficial responses following an adolescent’s suicide attempt. This difficulty may even indirectly affect the emotional fluctuations in the adolescent’s recovery process. Thus, the spectrum of emotional changes displayed by caregivers upon learning about a child’s suicidal behavior underscores the necessity for appropriate emotional support. This support is pivotal in assisting caregivers through this phase and mitigating the impact of such behavior on them. In a recent qualitative study, scholars like Weissinger emphasize the impact of adolescent suicide attempts on family caregivers [23]. Those who care for individuals with mental health issues or a history of suicide attempts face a heightened risk of developing similar conditions, as evidenced in another study [24]. The prolonged caregiving responsibilities can result in financial strain, particularly if caregivers must forgo or reduce their working hours to tend to a family member who has attempted suicide. In traditional Chinese culture, the occurrence of adolescent suicide behavior may evoke feelings of shame in family caregivers, leading to embarrassment or guilt, potentially heightening their risk of suicide [25]. Furthermore, the adolescent’s suicidal behavior itself constitutes a traumatic event, and family caregivers may undergo a nuanced grieving process, experiencing emotions of guilt, responsibility, or loss, which could contribute to post-traumatic stress disorder (PTSD) and other mental health issues [26].

While tending to adolescents who have attempted suicide, family caregivers encounter a myriad of challenges. Behaviors like smoking, homosexuality, and recurrent self-harm further complicate the caregiving landscape [27–29]. The study results underscore the difficulty family caregivers face in accepting the adolescent’s smoking behavior, presenting a cognitive challenge. Despite being tethered to traditional values, caregivers must recognize the individual differences in their child to prevent triggering emotional distress. When confronted with a child expressing homosexual thoughts, caregivers find it challenging to offer appropriate guidance and lack specific coping strategies. Furthermore, confronted with futile warnings regarding repetitive self-harm, caregivers experience a sense of helplessness and profound distress. Adolescent smoking may function as an avenue for emotional release, homosexuality as a distinctive mode of expressing emotions, and repetitive self-harm as a manifestation of internal anguish. However, within the confines of specific societal norms, these behaviors present formidable challenges for family caregivers. Concurrently, pervasive communication barriers present a significant challenge for family caregivers. The intricacies of gaining profound insights into the child’s inner world make it challenging for caregivers to effectively address existing issues, resulting in a weakened emotional connection with the adolescents. This not only hampers the recovery of the adolescents but also amplifies the psychological pressure on the caregivers. This suggests that caregivers lack effective strategies to navigate these challenges, rendering the task of caring for the child fraught with uncertainty. Therefore, during the hospitalization period, providing caregivers with scientific education on adolescent mental health, smoking, homosexuality, and self-harm issues. Organize regular seminars inviting mental health professionals, social workers, and experienced caregivers to share knowledge and discuss strategies for addressing adolescent behavioral issues. Establish a caregiver support network where they can share personal experiences, learn from one another, and provide emotional support. To facilitate a better understanding of the underlying motivations and management approaches for these behaviors. Furthermore, healthcare professionals can develop and provide educational manuals and promotional materials covering key topics such as adolescent mental health, the dangers of smoking, acceptance and support of diverse sexual orientations, and prevention of self-harm behaviors. These resources aim to provide family caregivers with insights into the motivations and emotional needs behind adolescent behavior, guiding them on how to better support and care for teenagers.

The French study on survival analysis of tree queues revealed a significant increase in the risk of subsequent suicide after an initial suicide attempt [30]. The interview outcomes from this study highlight that, when family caregivers inquire about adolescents’ remorse regarding prior suicide attempts, prevalent responses include expressions such as ‘’I would make the same choice if presented with another opportunity. ‘’ These responses are consistent with findings from existing international research [31], indicating that adolescents frequently harbor intense suicidal thoughts even after a suicide attempt. Caregivers express deep concern about suicidal thoughts or behaviors in adolescents, simultaneously conveying a feeling of helplessness in averting the reoccurrence of such behaviors. This underscores the considerable psychological burden placed on family caregivers. A path analysis study examining caregiving stress, attitudes toward suicide, and the caregiving competence of those caring for individuals with suicidal tendencies [32] suggests that caregivers encounter significant challenges in tending to individuals at risk of suicide. This encompasses elements of caregiving confusion and apprehension, shedding light on a deficiency in corresponding caregiving capabilities. This study illuminates that a significant majority, precisely two-thirds, of family caregivers harbor apprehensions concerning their children’s mental health challenges, previous suicidal behaviors, and ongoing psychiatric hospitalization records. They posit that these factors could cast adverse implications on the child’s forthcoming life, employment prospects, and even military service. In China, joining the military as a young person is considered a prestigious honor, but it requires meeting strict enlistment criteria. According to the conscription law, applicants must undergo comprehensive physical and mental examinations. Mental health assessments are particularly crucial, as soldiers need to possess strong psychological resilience to cope with various challenges. The Chinese military has established clear standards for mental health, and any psychological disorder that might hinder military duties could pose a barrier to selection. As a result, family caregivers worry that if their children have mental health issues, a history of suicidal behavior, or records of psychiatric hospital treatment, they might not meet the health requirements for enlistment, thus impacting their chances of joining the military. This underscores that caregivers’ anxiety transcends immediate crises, evolving into a protracted psychological distress necessitating increased support and intervention.

In a qualitative study on the family experiences of suicide attempt survivors in Ghana, researchers utilized an interpretative phenomenological research approach. Employing purposive sampling, they invited 10 families of suicide attempt survivors to participate in the study, which revealed three primary themes: Experiencing shame and stigma, Reactive affect, and Surviving the stress of attempt [33], it was discovered that caregivers faced a profound sense of stigma in the aftermath of such incidents. Echoing the outcomes of this investigation, suicide attempts not only exposed the individuals to societal stigmatization but also induced feelings of shame among family caregivers. Following attempted suicide by adolescents, caregivers hesitated to seek assistance, driven by concerns about societal stigma directed towards both the attempters and themselves. They tended to employ personalized coping mechanisms, such as concealment, avoidance, and confidentiality, as psychological and behavioral measures. The apprehension of shame associated with a child’s suicide attempt intensified the personal strain on caregivers, strained their social networks, heightened internal trauma, resulting in adverse mental health consequences. Interview findings additionally revealed that caregivers struggled to garner support from those closest to them, including friends and family, making hospitals the sole recourse for relief. Nevertheless, the term ‘’psychiatric hospital’’ carries a stigma for caregivers. Within the adolescent community, family caregivers hold a pivotal position in direct ‘’suicide monitoring’‘ [34], but due to the presence of shame, this monitoring role isn’t effectively fulfilled. Research findings suggest that the shame experienced by family caregivers becomes a motivating factor for them to avoid seeking external assistance [35, 36]. This sense of shame further diminishes their commitment to actively monitor the recovery process of adolescents, particularly those already grappling with mental health disorders. The withdrawal behavior of caregivers may heighten the patients’ feelings of shame, obstructing the effective fulfillment of the ‘’monitoring’’ role and thereby impeding recovery after a suicide attempt. Enveloped in shame, family caregivers find it challenging to access comprehensive support, impeding their capacity to facilitate positive recovery in adolescents.

To enhance the mental well-being of family caregivers managing the psychological strain of caring for adolescents with suicidal tendencies, proactive psychological interventions are essential. These interventions include tailored support, psychological counseling, and companionship services, alongside the establishment of support groups that facilitate information sharing and bolster psychological and social support. Seminars should be conducted to educate caregivers on adolescent suicide, aiming to alleviate anxiety, redirect self-blame, and provide tailored support. Family therapy and accurate sexual education are crucial for improving communication and dispelling misconceptions about homosexuality. Comprehensive suicide care education for caregivers, prior to patient discharge, should cover understanding suicide complexities, recognizing warning signs, creating secure home environments, and formulating no-suicide contracts. Additionally, insights into national policies and legal frameworks can empower caregivers to advocate for adolescent mental health. Post-hospitalization, healthcare professionals must offer enhanced, systematic support, including up-to-date information and forums for shared experiences. Open communication about the adolescent’s experiences, accurate information on suicide and self-harm, and education on patient rights and legal protections can reduce stigma and facilitate access to assistance, ultimately benefiting the adolescent’s holistic recovery.

### Limitations

The current study has several limitations in investigating the information requirements of family caregivers involved in caring for adolescents who have attempted suicide. Although we identified some key themes, the research did not encompass all the necessary information categories for caregivers. Specifically, we did not thoroughly extract and analyze caregivers’ specific needs concerning treatment management, access to community resources, comprehension of legal policies, pursuit of financial aid, and long-term strategizing. These needs are subject to change and evolve alongside the progression of adolescent treatment and the shifting adaptation of caregivers. Thus, the establishment of a comprehensive information and support infrastructure is imperative to address the continually evolving demands of family caregivers.

Influenced by Chinese culture, traditional values place a strong emphasis on maintaining family honor and dignity. As a result, some families may hesitate to openly discuss personal issues or challenges faced by family members, such as a child’s suicidal behavior. Additionally, cultural principles like ‘’respect for the elderly and cherishing the young’’ and ‘’concern for inequality rather than scarcity’’ can shape family members’ perspectives on suicide, prompting them to favor internal problem-solving over open dialogue or seeking outside help. Consequently, many family caregivers may be reluctant to participate in research or discuss their child’s suicidal behavior. Recognizing these cultural nuances is crucial when delivering information and support services, ensuring they are culturally sensitive to effectively address the diverse needs of caregivers. Future research should aim to address these challenges by gaining a comprehensive understanding of caregivers’ needs through expanded sample collection, in-depth thematic analysis, and the use of culturally informed methodologies to offer targeted and effective support.

### Implications for mental health nursing

This study holds significant implications for psychiatric nurses in hospital wards. Suicide is a major global mental health concern, and this behavior often faces stigmatization both domestically and internationally. In China, influenced by traditional Confucian moral thoughts, suicide is deemed shameful, leading to potential stigmatization of individuals engaging in such behavior and their family caregivers. Consequently, family caregivers of suicide attempt survivors struggle to access effective information support, with “psychiatric hospitals” being the primary source of assistance in China. It is evident that the support and information provided by psychiatric mental health nurses in hospital wards are crucial for the recovery of both caregivers and adolescents.

Currently, Chinese laws and policies lack specific provisions in addressing the stigma arising from suicide, indicating a need to establish relevant regulations and offer corresponding policy support to alleviate the adverse impact of stigma on family caregivers. Psychiatric specialized hospitals not only need to focus on patients’ psychological conditions but also must emphasize meeting the caregiving needs of family members, as this directly influences the patients’ recovery process. This research holds significant value for Chinese mental health nurses working in other countries, as it aids in gaining deeper insights into caregivers’ needs.

This study illuminates the emotional challenges and information requirements faced by Chinese family caregivers dealing with adolescent suicide attempts. These insights prompt psychiatric nurses to employ culturally sensitive approaches in clinical settings, offering tailored care plans and psychological support to better address caregivers’ needs. Additionally, the research highlights the significance of interdisciplinary collaboration, encouraging nurses to collaborate with other healthcare professionals to bolster support for family caregivers. Psychiatric nurses can utilize these research findings to advocate for policy reforms, enhance the overall support landscape for family caregivers, and integrate this knowledge into ongoing education for professional advancement. Through the establishment of effective assessment and feedback mechanisms, nurses can ensure that care services not only meet the actual needs of family caregivers but also respect their cultural backgrounds, facilitating more meaningful interventions and comprehensive care in the mental health domain.

## Conclusion

Faced with adolescents’ attempted suicide, family caregivers undergo intricate emotional responses. Throughout the caregiving process, they confront various challenges, feeling challenged to make the right decisions to guide the adolescents and apprehensive that their misguidance might trigger further suicide attempts. Compounded by the constraints of societal values in our country, caregivers are reluctant to seek external help, fearing potential stigmatization. Healthcare professionals should pay attention to the information gaps among family caregivers during their time in the hospital, seek solutions for them, and provide multifaceted support and assistance to alleviate the caregiving burden, thereby fostering the recovery of the adolescents. Helping caregivers understand and access various community resources, such as community centers, volunteer groups, and other available assistance. Establishing interdisciplinary teams comprised of doctors, nurses, social workers, and psychologists to deliver comprehensive services and support to caregivers. Providing easily understandable educational materials and manuals to aid caregivers in comprehending their loved ones’ illnesses and caregiving techniques. Collaborating with professionals to devise personalized caregiving plans for each family caregiver, considering their specific needs and circumstances.

### Relevance statement

In the care of adolescents who have attempted suicide, family caregivers play a pivotal role, shouldering significant responsibilities. However, research on the information needs of family caregivers in the context of caregiving for suicidal adolescents within the Chinese cultural framework is relatively scarce. This study aims to delve into the specific challenges faced by family caregivers in China when caring for adolescents who have attempted suicide, providing a more comprehensive understanding of their needs. The findings indicate that family caregivers encounter numerous difficulties during the caregiving process, necessitating support with relevant information to assist them in coping with challenges. Nurses in the psychiatric wards of specialized mental health hospitals bear the responsibility of offering information support and guidance to family caregivers of adolescents who have attempted suicide, alleviating their concerns when confronted with unknown situations. Currently, research in this field in China is still in its early stages, and mental health hospitals need to invest time and resources to establish an effective platform for providing information support to caregivers.

### Electronic supplementary material

Below is the link to the electronic supplementary material.

Supplementary Material 1

## Data Availability

The dataset used and analyzed during the current study is available from the corresponding author on reasonable request.
