# Supplementary material for: Caregiving information needs of family caregivers of adolescent patients with suicide attempts: a qualitative study in China
Source: BMC Nurs. 2024 Jun 28;23:445. doi: 10.1186/s12912-024-02120-7 (PMC11212159; doi:10.1186/s12912-024-02120-7)
Supplement: Supplementary file 1 — Supplementary Material 1 [file 12912_2024_2120_MOESM1_ESM.docx]

**Supplementary Table 1：COREQ**

| **No** | **Item** | **Guide questions/description** | **Pagination** |
| --- | --- | --- | --- |
| **Domain 1: Research team and reflexivity** |  |  |  |
| Personal Characteristics |  |  |  |
| 1. | Interviewer/facilitator | Which author/s conducted the interview or focus group? | First author |
| 2. | Credentials | What were the researcher's credentials? E.g. PhD, MD | Master of Medicine |
| 3. | Occupation | What was their occupation at the time of the study? | Master's Degree, Associate Professor |
| 4. | Gender | Was the researcher male or female? | Female |
| 5. | Experience and training | What experience or training did the researcher have? | Qualitative research, the first author has been published in China Beida Chinese core two. |
| Relationship with participants |  |  |  |
| 6. | Relationship established | Was a relationship established prior to study commencement? | Yes |
| 7. | Participant knowledge of the interviewer | What did the participants know about the researcher? e.g. personal goals, reasons for doing the research | P7 |
| 8. | Interviewer characteristics | What characteristics were reported about the interviewer/facilitator? e.g. Bias, assumptions, reasons and interests in the research topic | P6 |
| **Domain 2: study design** |  |  |  |
| Theoretical framework |  |  |  |
| 9. | Methodological orientation and Theory | What methodological orientation was stated to underpin the study? e.g. grounded theory, discourse analysis, ethnography, phenomenology, content analysis | P6 |
| Participant selection |  |  |  |
| 10. | Sampling | How were participants selected? e.g. purposive, convenience, consecutive, snowball | P6 |
| 11. | Method of approach | How were participants approached? e.g. face-to-face, telephone, mail, email | P7 |
| 12. | Sample size | How many participants were in the study? | P8 |
| 13. | Non-participation | How many people refused to participate or dropped out? Reasons? | 3 refused to participate. Because didn't really want people to know too much about their family and wanted to protect their children. |
| Setting |  |  |  |
| 14. | Setting of data collection | Where was the data collected? e.g. home, clinic, workplace | P8 |
| 15. | Presence of non-participants | Was anyone else present besides the participants and researchers? | P8 |
| 16. | Description of sample | What are the important characteristics of the sample? e.g. demographic data, date | P5 |
| Data collection |  |  |  |
| 17. | Interview guide | Were questions, prompts, guides provided by the authors? Was it pilot tested? | P5 |
| 18. | Repeat interviews | Were repeat interviews carried out? If yes, how many? | Repeat interviews were not conducted because this study received key information in each of the interviews. |
| 19. | Audio/visual recording | Did the research use audio or visual recording to collect the data? | Use a Chinese app called "YouDao YunNote" to record audio |
| 20. | Field notes | Were field notes made during and/or after the interview or focus group? | P7 |
| 21. | Duration | What was the duration of the interviews or focus group? | P7 |
| 22. | Data saturation | Was data saturation discussed? | P6 |
| 23. | Transcripts returned | Were transcripts returned to participants for comment and/or correction? | P7 |
| **Domain 3: analysis and findings**z |  |  |  |
| Data analysis |  |  |  |
| 24. | Number of data coders | How many data coders coded the data? | P8 |
| 25. | Description of the coding tree | Did authors provide a description of the coding tree? | Not provided |
| 26. | Derivation of themes | Were themes identified in advance or derived from the data? | It is based on the content of the interviews derived after the interviews were organized by the researcher and integrated to come up with themes. |
| 27. | Software | What software, if applicable, was used to manage the data? | P7 |
| 28. | Participant checking | Did participants provide feedback on the findings? | P7 |
| Reporting |  |  |  |
| 29. | Quotations presented | Were participant quotations presented to illustrate the themes / findings? Was each quotation identified? e.g. participant number | P11 |
| 30. | Data and findings consistent | Was there consistency between the data presented and the findings? | Concordance |
| 31. | Clarity of major themes | Were major themes clearly presented in the findings? | P10 |
| 32. | Clarity of minor themes | Is there a description of diverse cases or discussion of minor themes? | P10 |

Consolidated criteria for reporting qualitative studies (COREQ):32-item checklist
